# A morphological traits dataset of Heteroptera sampled in biodiversity priority areas of Southwest China

**DOI:** 10.1038/s41597-024-03556-x

**Published:** 2024-06-26

**Authors:** Shutong Gao, Wenbo Yu, Ting Tian, Zhixing Lu, Xiang Zhang, Qiao Li, Youqing Chen

**Affiliations:** 1 Institute of Highland Forest Science, Chinese Academy of Forestry, Yunan Kunming, 650224 China; 2 Key Laboratory of Breeding and Utilization of Resource Insects of National Forestry and Grassland Administration, Yunnan Kunming, 650224 China; 3 Nanjing Forestry University, Jiangsu Nanjing, 210037 China; 4 Southwest Forestry University, Yunan Kunming, 650224 China

**Keywords:** Ecology, Ecology

## Abstract

Functional traits reveal the adaptive strategies of species to their environment, and are relevant to the formation of communities, the function of ecosystems, and the mechanisms underlying biodiversity. However, trait databases have not been established for most biological taxa, especially for insects, which encompass a vast number of species. This study measured the morphological traits of 307 species of Heteroptera insects collected in 2019 from the “Xishuangbanna Priority Conservation Area” in Southwest China using sweep netting and light trapping methods. This study provides a dataset for 307 Heteroptera species, comprising 34 morphometric measurements and 17 morphological traits. The dataset contains information on species sex, abundance, and the average, maximum, and minimum values of traits. This dataset facilitates an enhanced understanding of the functional traits and ecological associations of Heteroptera insects and offers opportunities for exploring a more diverse range of research topics.

## Background & Summary

Research methodologies based on functional trait measurements offer a robust framework for comparing the ecological differences among species across various scales and for describing their community structure characteristics^1–3^. These methodologies hold promising applications in unveiling the mechanisms of species coexistence within communities^4,5^. There is a strong correlation between environmental filtering and the functional traits of species; the loss of traits often reflects the local biological community’s response to environmental changes^1,3^. Therefore, studying the functional traits of organisms within a community can provide insights into the assembly patterns of the community^6^, aiding in the understanding of the correlation between the environment and the functional structure of biological communities^3,7^. This, in turn, offers novel insights into the mechanisms underlying community formation. By focusing on functional traits, researchers can not only elucidate the factors contributing to the formation of insect communities but also identify the driving forces behind the evolution of community structures^8^.

Over the past few decades, regional and global trait datasets for various biological taxa have been sequentially established, with traits becoming an increasingly utilized research element within the fields of ecology, biodiversity conservation, ecosystem restoration, and landscape management^1,5,9,10^. Despite global efforts to construct trait databases, the majority of existing databases are primarily focused on plants, birds, and amphibians, representing relatively small taxonomic groups^11–15^. In contrast, focusing on insect taxa presents a formidable challenge due to the vast number of species, making the collection of research-valuable trait data a daunting task. Additionally, much of the existing research on insect traits is based on characteristics derived from literature^16–20^, such as trophic-defined groups or binary long- and short-winged groups, which are discrete and lose critical information. This is suboptimal for analyzing the impact of abiotic environmental filters on the composition of functional communities^1,21,22^. Therefore, it is imperative to measure accurate and comprehensive morphological characteristics of insects and establish a morphological trait database.

The Heteroptera represents a vast group within the hemimetabolous insects, with over 45,000 species identified globally^23^. Characterized by their extensive distribution and morphological diversity, they serve as ideal subjects for ecological studies^21,24–29^. Gossner *et al*. established a database containing mean values of 23 morphometric measurements for 179 heteropteran species collected from German grasslands^30^. This database laid the foundation for the development of a trait database for the Heteroptera, instilling confidence in subsequent research efforts. To expand upon this existing database, we measured 34 morphological characteristics of 307 Heteroptera species collected from a biodiversity priority area in China. To ensure the standardization and availability of trait datasets, this study employed the same measurement techniques and trait classification methods utilized in the morphological trait database of Heteroptera from German grasslands^30^. Furthermore, the value of measuring diverse morphological traits for further analysis of functional responses has been demonstrated by individual studies on single taxonomic groups, such as the effect of mantis hind tibia width^31^ and lepidopteran wing area^32,33^ on flight performance. Therefore, acquiring additional trait information is necessary to detect the response of arthropod communities to environmental changes or the impact of trait variation on ecosystem function. Thus, our study incorporated additional measurements of features such as the tibia width and wing area, enhancing the dataset’s comprehensiveness.

## Methods

### Sites

**Fig. 1:**
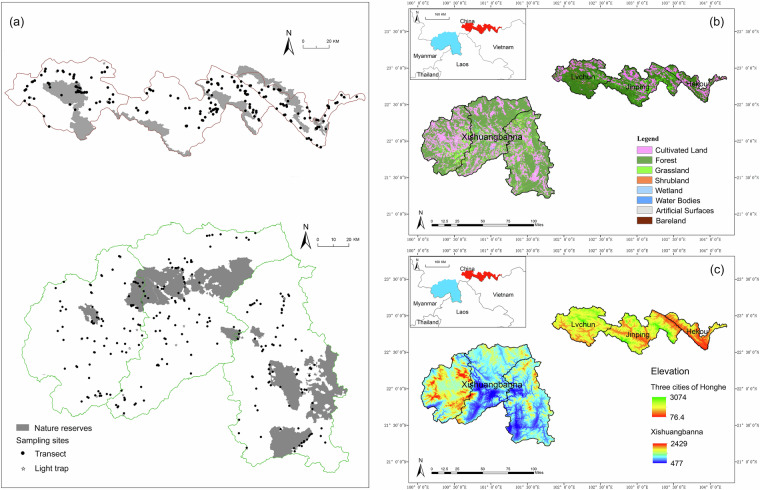
Study sites. (**a**) Sampling sites; (**b**) Land-cover map of study sites at 30 m resolution; (**c**) Elevation.

This study conducted sampling within two areas, Xishuangbanna Prefecture and three counties of Honghe Prefecture, located in the “Xishuangbanna Priority Conservation Area (42,858 km^2^, including six national nature reserves)” in the southwest region of China. The sampling sites included five national nature reserves (Fig. 1a). The study areas have been subject to long-term human activity, resulting in a complex landscape structure interlaced with urban areas, forests, and farmlands^34,35^ (Fig. 1b).

#### Xishuangbanna

Xishuangbanna Prefecture, located within Yunnan Province of China, encompasses an area of 19,124.5 km^2^, including two national nature reserves, with an elevation range from 477 meters to 2,429 meters (Fig. 1c). This region is characterized by a tropical monsoon climate.

#### Three cities in Honghe

Lüchun, Jinping, and Hekou are three counties located in the Honghe Prefecture of Yunnan Province, China, encompassing a total area of 8,105.86 km^2^, which includes three national nature reserves. Situated in a low-latitude subtropical plateau with a moist monsoon climate, their elevation ranges from 76.4 meters to 3,074 meters (Fig. 1c).

### Experimental and sampling design

In this study, the research area was delineated into grid units of 10 kilometers each, with 1 to 2 sampling sites selected per working grid. At each sampling site, three transects of 100 meters in length were established. From July to October 2019, insect samples were collected using sweep netting and light trapping methods. Each study area underwent two rounds of sampling. All specimens collected were preserved in 75% ethanol solution and transported back to the laboratory for identification.

### Traits acquisition and classification

**Fig. 2:**
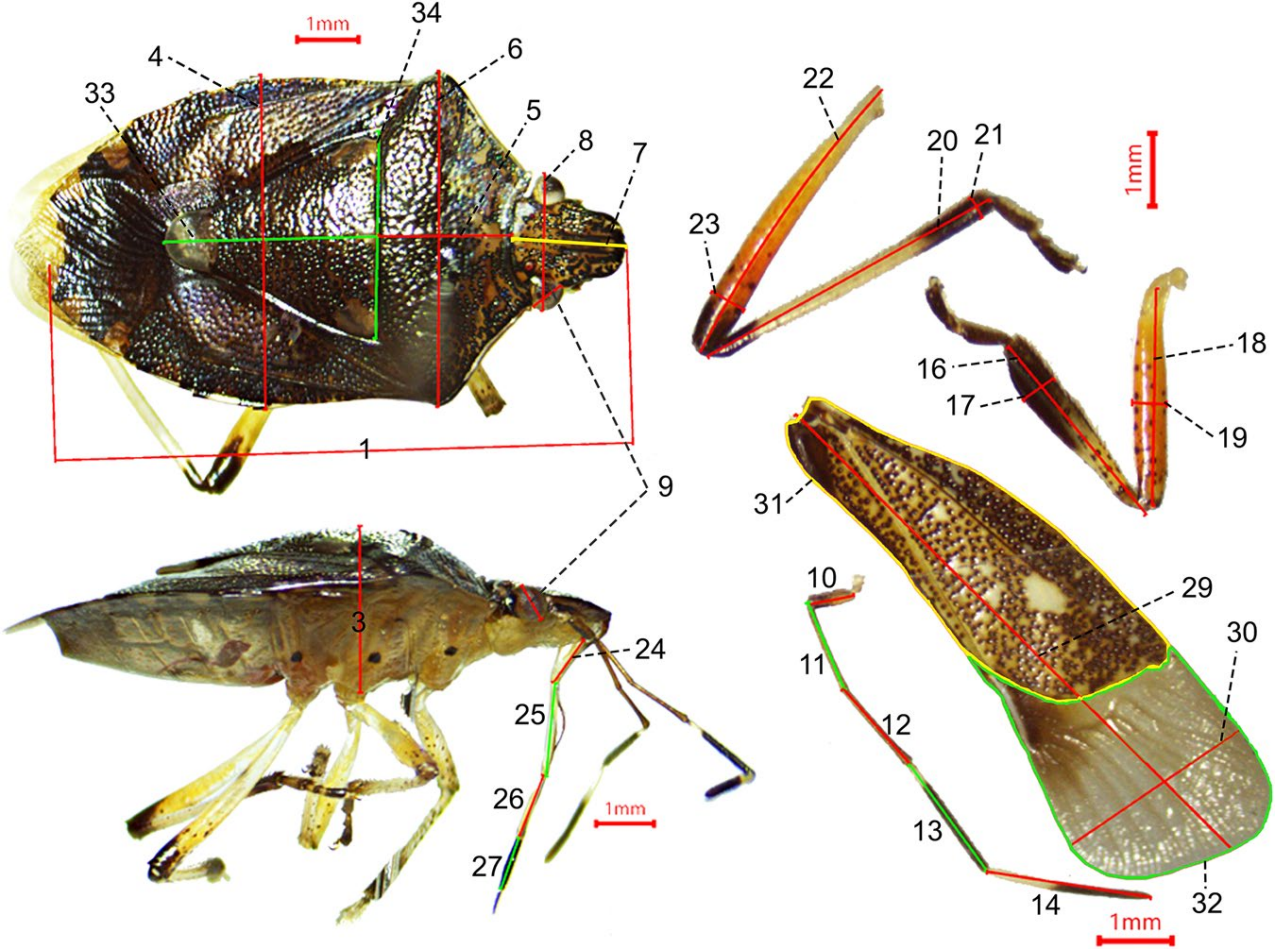
Illustration of morphometric measurements taken on each specimen. The numbers correspond to morphometric measurement codes in Table 1.

We employed a stereoscopic dissecting microscope equipped with a ToupCam digital camera (1/1.8” Sony Exmor CMOS sensor) and the ToupTek ToupView 3.7 software to take morphometric measures, the measurement precision is 0.01 mm. A total of 34 morphometric measurements were performed on each individual, with measurement locations and definitions following previous studies^23,30,36^. For details see Table 1 and Fig. 2.

**Table 1:** Overview of all variables in data set 1: “HeteropteraMorphometricTraits_1.csv”.

code	Variables	Type	Description
	Family	factor	Taxonomic status of species
	Chinese name	factor	Chinese name of the species
	SpeciesID	factor	species name accepted with the species standardization protocol
	Sex	factor	Sex of measured specimen: m = male, f = female
	Numbers	numeric	The total number of species
1	Body length	numeric	From the tip of the head to the end of the abdomen^30,36^
2	Body width	numeric	Widest part of the body^30^
3	Body height	numeric	Thickest part of the body^30^
4	Abdomen width	numeric	Widest part of the abdomen^30,36^
5	Thorax length	numeric	Length of the central axis of the Thorax^30,36^
6	Thorax width	numeric	Widest part of the pronotum^30^
7	Head length	numeric	Longest part of the head^36^
8	Head width	numeric	Widest part of the head including eyes^30^
9	Eye width	numeric	Widest part of the left eye (dorsal / side view)^30^
10	Antenna Seg1	numeric	Length of first antenna segment^30,36^
11	Antenna Seg2	numeric	Length of second antenna segment^30,36^
12	Antenna Seg3	numeric	Length of third antenna segment^30,36^
13	Antenna Segt4	numeric	Length of fourth antenna segment^30,36^
14	Antenna Seg5	numeric	Length of fifth antenna segment^30^
15	Antenna length	numeric	Length of the antenna including all segments^30^
16	Front-Tibia length	numeric	Length of the tibia of the foreleg^30^
17	Front-Tibia width	numeric	Width of the tibia of the foreleg^30^
18	Front-Femur length	numeric	Length of the femur of the foreleg^30^
19	Front-Femur width	numeric	Width of the femur of the foreleg^30^
20	Hind-Tibia length	numeric	Length of the tibia of the hind leg^30^
21	Hind-Tibia width	numeric	Width of the tibia of the hind leg^30^
22	Hind-Femur length	numeric	Length of the femur of the hind leg^30^
23	Hind-Femur width	numeric	Width of the femur of the hind leg^30^
24	Rostrum Seg1	numeric	Length of first rostrum segment^30,36^
25	Rostrum Seg2	numeric	Length of second rostrum segment^30,36^
26	Rostrum Seg3	numeric	Length of third rostrum segment^30,36^
27	Rostrum Seg4	numeric	Length of fourth rostrum segment^30^
28	Rostrum length	numeric	Length of the rostrum including all segments^30^
29	Wing length	numeric	Longest part of the forewing^30,36^
30	Wing width	numeric	Widest part of the forewing^30^
31	Leathery area	numeric	The sum of the corium and clavus areas (except membranous corium)^23,39,40^
32	Membranous area	numeric	Area of the membrane (some species include membranous corium)^23,39,40^
33	Scutellum length	numeric	Longest part of the scutellum^36^
34	Scutellum width	numeric	Widest part of the scutellum^36^

For all sampled species within the Heteroptera, species with a sufficient number of individuals were subject to the morphological measurement of at least five specimens to incorporate intraspecific variability into our measurement of species characteristics. The sampling effort aimed to include both female and male individuals, with the sex ratio dependent on the sampling conditions. Individuals of different sexes were selected from the study samples and mounted on a work board using entomological pins to ensure the insect’s body remained parallel to the microscope objective. We disassembled the insect’s forelegs, hindlegs, antennae, and forewings for precise measurement, ensuring that the parts being measured were parallel to the objective to guarantee the reliability of the measurements. For species with an adequate number of samples, individuals with partial damage were excluded from measurement. For species with fewer than five individuals, only measurements of the damaged parts were omitted.

We identified 17 morphometric traits representing five characteristic categories^30^, including body size (body length, volume^37,38^), dispersal capability (relative wing length, ratio of membranous to leathery wing area, front tibia shape, hind tibia shape^31^, relative length of the hind femur^39^), resource utilization in feeding (relative rostrum length^40–43^, shape of the front femur^44^), habitat utilization (body size, relative length of the scutellum), and orientation ability (relative antenna length, relative eye size^45^). In addition to traits with demonstrated functionality, we incorporated several predictive traits, species with a greater proportion of membranous wing area are expected to exhibit enhanced dispersal capabilities^32,33^; anticipating the shape of the front tibia and hind tibia are related to dispersal capabilities, with species possessing broader tibiae exhibiting stronger dispersal abilities^31^; and significant morphological variation in the scutellum across different species of Heteroptera, its relative length is predicted to be associated with habitat utilization^46–48^. For more details see Table 2.

**Table 2:** Overview of all variables in data set 2: “HeteropteraMorphometricTraits_2.csv”.

Trait category	Morphometric trait	Type	Description
	Family	factor	Taxonomic status of species
	Chinese name	factor	Chinese name of the species
	SpeciesID	factor	species name accepted with the species standardization protocol
	Sex	factor	Sex of measured specimen: m = male, f = female
	Numbers	numeric	The total number of species
Body size^37,38^	Body length	numeric	Total length
	Body volume	numeric	Body length × body width × body height
Dispersal ability	Rel. Forewing length^32,33^	numeric	Forewing length / body length
	Area ratio of the forewing^32,33^	numeric	Membrane area / Coriaum & clavus area
	Hind-Femur shape^30^	numeric	Femur length / femur width
	Hind-Tibia shape^31^	numeric	Tibia length / tibia width
	Front-Tibia shape^31^	numeric	Tibia length / tibia width
	Rel. Hind-Femur length^39^	numeric	Femur length / body length
	Rel. Hind-Tibia length^30^	numeric	Tibia length / body length
Feeding resource use	Rel. Rostrum length^40–43^	numeric	Rostrum length / body length
	Front-Femur shape^44^	numeric	Femur length / femur width
	Rel. Front-Femur length^30^	numeric	Femur length / body length
	Rel. Front-Tibia length^30^	numeric	Tibia length / body length
Habitat use	Body shape^37,38^	numeric	Body length / body width
	Rel. Scutellum length^46–48^	numeric	Scutellum length / body length
Orientation	Rel. Eye size^45^	numeric	Eye width / head width
	Rel. Antenna length^30^	numeric	Total antenna length / body length

The 17 morphological traits were calculated from the 34 morphometric measurements (see Table 1). The detailed calculation formulas can be found in the “Description” column of Table 2. All computations were conducted using Microsoft Excel software.

## Data Records

This dataset is a species-level data set of 34 morphometric measures from 4346 specimens of 307 species of Heteroptera. For 299 species (about 97%), at least five individuals were measured for each species. The comprehensive dataset is constituted by three discrete files: two of which are formatted in ‘.csv’ (Unicode UTF-8), and one in PDF format. All files are accessible via figshare^49^. Rows denote unique species records, while columns correspond to the variables provided.

### HeteropteraMorphometricTraits_1

This file encompasses morphometric measurements conducted on both male and female specimens across 307 species within the Heteroptera, comprising 34 distinct morphological measurements. The file presents the mean, minimum, and maximum values of these measurements for the collective specimens of each species, as well as for individual female and male specimens, with all dimensions denoted in millimeters. Additionally, the dataset includes information on the number of individuals measured, as well as taxonomic data at the order and family levels.

### HeteropteraMorphometricTraits_2

The morphological traits in the file, designated as HeteropteraMorphometricTraits_2 were derived through calculations based on 34 morphometric measurements (see Table 1) taken from specimens of 307 species within the Heteroptera. For each species, average, minimum, and maximum values were computed for the total specimens measured, as well as separately for female and male individuals, with all measurements presented in millimeters. Additionally, the file encompasses information on the number of individuals, as well as their respective orders and families.

### HeteropteraFigures

This study presents a total of 310 photos of all measured species for verification purposes and to ensure the future usability of the data.

## Technical Validation

All data contained within this dataset were provided by the authors. Field methods followed a standardized protocol (Supplementary Tables S1 and S2). To ensure comparability, future data reuse and synthesis, both the primary measurement methods and classification schemes for the traits were consistent with those of Gossner *et al*.^30^. Species identification was cross-checked by different taxonomic experts and at random by barcoding. Although the measurement of morphological traits involves a minimal degree of subjective intervention, the measurement of all trait data was conducted by the same individual to minimize subjective bias.

This study examined whether data for each trait contained abnormal values or outliers. In most instances, these issues were addressed by inspecting images of the species with attached scales and the specimens themselves. All specimens are stored in the Institute of Highland Forest Science, Chinese Academy of Forestry.

## Usage Notes

The information allocated on the *Figshare* repository is a static version of the data last reviewed on June 2024, further updates and refinements planned for release within the same Figshare project, but the DOI could be different. A report record will be also included to control further updates. Anticipated future expansions include the incorporation of ecological trait dataset and site-specific information. Traits can be univocally linked to each species included in the latter datasets (or others) using the species Latin name.

### Supplementary information

Supplementary information of Morphometric measures of Heteroptera sampled in China

## Data Availability

No custom code was used to generate or process the data in this study.
