# Supplementary material for: A morphological traits dataset of Heteroptera sampled in biodiversity priority areas of Southwest China
Source: Sci Data. 2024 Jun 26;11:694. doi: 10.1038/s41597-024-03556-x (PMC11208582; doi:10.1038/s41597-024-03556-x)
Supplement: Supplementary file 1 — Supplementary information of Morphometric measures of Heteroptera sampled in China [file 41597_2024_3556_MOESM1_ESM.pdf]

Supplementary information of Morphometric measures of Heteroptera sampled in China

Supplementary Table S1 The record sheet used for transects in the field
Supplementary Table S2 The record sheet used for light trapping in the field

Supplementary Table S1 The record sheet used for transects in the field

Site No.		Site name	Province, county, township (town), village				
Start	Latitude			Longitude			
End	Latitude			Longitude			
Elevation		Data		Time		Team	
Weather		Temp.		Humidity		micro-topo graphy	
Vegetation type		Habitat type		Disturbance type		Disturbance strength	
Grid No.			Habitat photos No.				
Specimen No.	Collection methods (net sweeping, hand picking, shaking)		Number	photos No.		Remarks	

Supplementary Table S2 The record sheet used for light trapping in the field

Site No.		Site name	Province, county, township (town), village				
Data		Start time		End time		Duration	
Elevation		Latitude		Longitude		Team	
Weather		Temp.		Humidity		micro-topo graphy	
Vegetation type		Habitat type		Disturbance type		Disturbance strength	
Grid No.			Habitat photos No.				
Specimen No.		Number		Photos No.		Remarks	
